# Preoperative Ketamine Gargle for Prevention of Postoperative Sore Throat After Tracheal Intubation in Adults: A Meta-Analysis

**DOI:** 10.1155/prm/7622696

**Published:** 2025-01-29

**Authors:** Saihao Fu, Mengrong Miao, Jing Bian, Yunxiang Fu, Jiaqiang Zhang, Mingyang Sun

**Affiliations:** ^1^Department of Anesthesiology and Perioperative Medicine, People's Hospital of Zhengzhou University, Henan Provincial People's Hospital, Zhengzhou 450003, China; ^2^Department of Anesthesiology and Perioperative Medicine, People's Hospital of Henan University, Henan Provincial People's Hospital, Zhengzhou 450003, China; ^3^Department of Anesthesiology and Perioperative Medicine, Henan Provincial People's Hospital, People's Hospital of Zhengzhou University, People's Hospital of Henan University, Zhengzhou 450003, China

## Abstract

**Objective:** This meta-analysis aims to evaluate the impact of preoperative ketamine gargle on postoperative throat pain in patients undergoing general anesthesia with endotracheal intubation (ETI).

**Methods:** A comprehensive search was conducted in databases including PubMed, Cochrane Library, Web of Science, ScienceDirect, Scopus, ClinicalTrials.gov, and others. Data analysis was performed using RevMan 5.4 and Stata Statistical Software 18 (StataCorp., Texas., United States of America). Odds ratio with 95% confidence interval (CI) and mean difference were calculated for outcomes: incidence of postoperative 0, 2, 4, 8, and 24 h sore throat and anesthesia time. The overall certainty of evidence was evaluated with the Grading of Recommendations Assessment, Development and Evaluation (GRADE) approach, with trial sequential analysis (TSA) performed to establish implications for further research.

**Main outcome:** A total of ten RCTs involving 593 patients were included in the analysis. The results demonstrated a significant reduction in the incidence of postoperative sore throat at 0, 2, 4, 8, and 24 h after the operation (0 h: OR: 0.14; 95% CI: 0.04–0.47; *p*=0.002; *I*^2^ = 67%; 2 h: OR: 0.30; 95% CI: 0.17–0.52; *p* < 0.0001; *I*^2^ = 31%; 4 h: OR: 0.32; 95% CI: 0.20–0.52; *p* < 0.00001; *I*^2^ = 0%; 8 h: OR: 0.40; 95% CI: 0.23–0.70; *p*=0.001; *I*^2^ = 29%; 24 h: OR: 0.36; 95% CI: 0.25–0.51; *p* < 0.00001; *I*^2^ = 0%) in patients who received ketamine gargle compared to those who received a placebo. In addition, our meta-analysis indicated that ketamine gargle did not result in a reduction in anesthesia time (min) (MD: −1.16; 95% CI: −6.44–4.11; *p*=0.67).

**Conclusion:** Our meta-analysis demonstrated the efficacy of prophylactic ketamine gargle in reducing the incidence of POST across all studied time intervals in patients requiring tracheal intubation of general anesthesia compared to placebo.

## 1. Background

Postoperative sore throat (POST) is a known complication of endotracheal intubation (ETI) under general anesthesia, with an incidence ranging from 28% to 80% [1]. Although POST is self-limiting [2]. it can lead to postoperative complications and significant discomfort for patients. Various nonpharmacological and pharmacological methods have been employed to alleviate POST.

Among nonpharmacological approaches, methods such as using smaller endotracheal tubes, lubricating the endotracheal tube with water-soluble gel, adequate relaxation prior to intubation, gentle suctioning of the oropharynx, minimizing cuff pressure, and deflating the cuff completely before extubation have been shown to reduce the incidence of POST [3]. On the other hand, pharmacological measures include inhalation of steroids and other drug gargles. Ionotropic N-methyl-D-aspartate (NMDA), alpha-amino-3-hydroxy-5-methyl-4-isoxazolepropionic acid (AMPA), and kainate receptors are present in the central and peripheral nervous systems [4]. Studies have suggested that activation of these receptors can contribute to nociceptive behavior and inflammatory pain [4, 5]. Furthermore, experimental research has demonstrated that peripherally administered NMDA receptor antagonists are involved in the analgesic and anti-inflammatory cascade response mediated by opioid receptors and NMDA receptor antagonists located in the oral and upper respiratory mucosa, as well as the interaction with cytokine production, inflammatory cell regeneration, and inflammatory mediators [4, 6–10].

However, there is currently conflicting evidence regarding whether ketamine (an NMDA receptor antagonist) gargle can reduce throat pain in patients after ETI [11, 12]. The aim of this study is to explore the potential of ketamine gargle in reducing throat pain in patients after ETI through the meta-analysis.

## 2. Method

### 2.1. Study Design and Registration

The protocol for this study was registered in the International Prospective Register of Systematic Reviews (CRD: 42024517271). The reporting of this study followed the guidelines outlined in the Preferred Reporting Items for Systematic Reviews and Meta-Analysis (PRISMA) to ensure comprehensive and transparent reporting (Supporting 1) [13].

### 2.2. Study Selection Criteria

Our study had the following inclusion criteria: (1) patients who underwent tracheal intubation under general anesthesia, (2) intervention with ketamine gargle, (3) study designs that included randomized controlled trials (RCTs), (4) the primary outcome being sore throat at 24 h after the operation, and (5) assessment of sore throat using a four-point scale tool (0: no sore throat; 1: mild sore throat [complains of sore throat only on asking]; 2: moderate sore throat [complains of sore throat on his/her own]; and 3: severe sore throat [change of voice or hoarseness, associated with throat pain]). The exclusion criteria were: (1) absence of a placebo control group, and (2) publications not in the English language.

### 2.3. Search Strategy

A comprehensive literature search was conducted by a researcher (FSH) in the following electronic databases from their inception: PubMed, Cochrane Library, Web of Science, ScienceDirect, Scopus, and ClinicalTrials.gov. The searches were performed on November 11, 2023. The search keywords are as follows: “postoperative sore throat,” “ketamine gargle,” “tracheal intubation.” Only studies written in English and involving human subjects were included. In addition, an additional search was conducted on PubMed and Google Scholar to identify articles that investigated the use of ketamine gargle in relation to POST. The reference lists of included articles were manually searched to identify any potentially missed studies from the systematic search (Search strategy see Supporting 2).

### 2.4. Study Selection and Data Extraction

Two reviewers (FSH and BJ) used the inclusion criteria to independently screen the titles and abstracts in the Rayyan systematic review application. Full-text studies were assessed for inclusion by two reviewers (FSH and BJ). Disagreements regarding the inclusion of abstracts and full-text articles were resolved through discussion among another reviewer (FYX) and the senior author (MYS) [14].

Three reviewers (FSH, BJ, and FYX) independently extracted data from the approved full-text studies. Extracted data consisted of study name, year of publication, participants' demographics, study design, surgery type, incidence of sore throat within 24 h (0, 2, 4, 8, and 24 h) after surgery, doses of ketamine used, POST scoring tool, anesthesia time, postoperative analgesia, and size of the tracheal tube.

### 2.5. Quality Assessment of Studies

Study quality was assessed by two investigators (FSH and BJ) based on the type of study. For RCTs, the Cochrane Collaboration's tool was utilized to evaluate bias across six domains: selection bias, performance bias, detection bias, attrition bias, reporting bias, and other bias [15]. Overall certainty of evidence was evaluated with the Grading of Recommendations Assessment, Development and Evaluation (GRADE) approach using the GRADEpro Guideline Development Tool (Software). McMaster University and Evidence Prime, 2024. Available from gradepro.org. Trial sequence analysis (TSA) was conducted to establish the effect of sample size on the study's further research. The primary indicators of study quality included clear identification of the study population, outcomes, and outcome assessment, no selective loss of patients during follow-up, and identification of important confounders and/or prognostic factors. Any conflicts were resolved by a third reviewer (FYX).

### 2.6. Data Analysis

We utilized Cochrane Review Manager Version 5.4 to conduct the meta-analysis employing the random effects model or the fixed effects model and statistical significance was determined at the 2-sided *p* < 0.05 level for all outcomes. When *I*^2^ was < 50%, we used the fixed effects model, otherwise, we used the random effects model. The odds ratios (ORs) (OR < 1, indicating that ketamine gargle is a protective factor) and mean difference (MD) were calculated for dichotomous and continuous outcomes, respectively [16]. 95% confidence interval (CI) (CI indicated the degree to which the true value of this parameter has a certain probability of falling around the measurement result) was also calculated. In cases where continuous outcomes were reported as medians with interquartile range, we converted them to means and standard deviations using the method proposed by Wan et al. [17] Statistical heterogeneity was assessed using the *I*^2^ statistic. Visual analysis of funnel plots and meta-regression were used to assess publication bias. Sensitivity analysis and meta-regression were performed using Stata Statistical Software 18 (StataCorp., Texas, United States of America).

## 3. Results

### 3.1. System Retrieval

**Figure 1:**
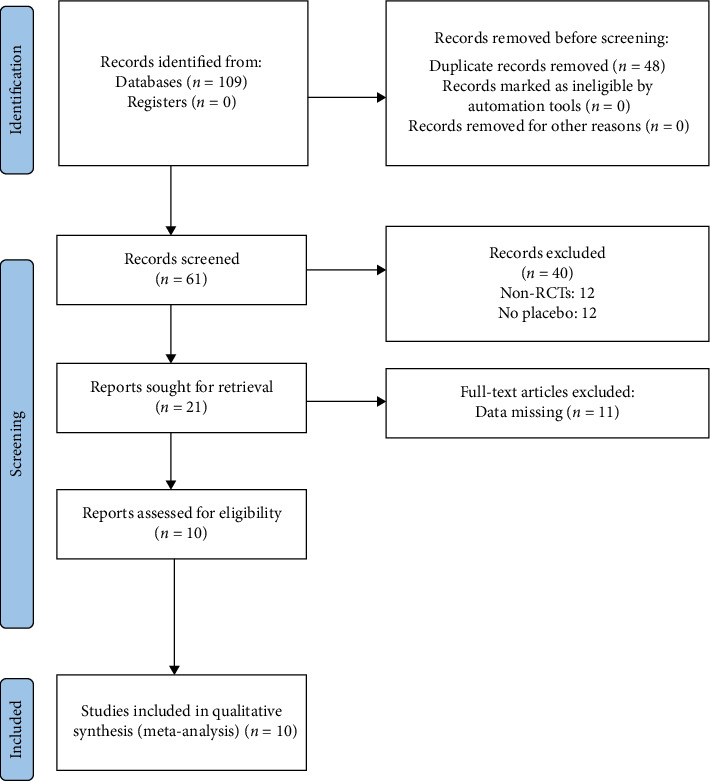
Screening flowchart.

A total of 109 studies were identified, but only 10 RCTs met the inclusion criteria and were included in our study [1–3, 11, 12, 18–22]. Among the excluded studies, 48 were excluded due to duplication. After reviewing the titles and abstracts, 40 studies were excluded. An additional 11 studies were excluded after a full-text examination. Specific reasons for exclusion can be found in Figure 1.

### 3.2. Basic Characteristics of Included Studies

A total of 593 adults (male/female: 295/298) with ASA Grade I-II were included in this study. The participants consisted of adults undergoing various types of surgeries, including pelvic/abdominal elective surgery, septorhinoplasty, abdominal and orthopedic surgery, ear surgeries, and elective surgery of unspecified types. Among the 10 studies included, 5 studies involved gargling 50 mg of ketamine in 29 mL of normal saline for 30 s [2, 3, 12, 18, 20], 3 studies used 40 mg of ketamine dissolved in 30 mL of normal saline for 30 s [11, 19, 21], 1 study utilized gargling 50 mg of ketamine in 30 mL of normal saline for 30 s [22], and 1 study involved gargling 50 mg of ketamine in 29 mL of normal saline for 40 s [1]. All included studies employed the 4-point scale to evaluate POST (Supporting Table 1 in Supporting 3).

#### 3.2.1. Main Outcome: Incidence of Sore Throat at 24 h After Operation

**Figure 2:**
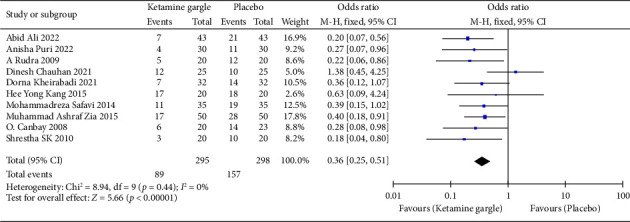
Forest plot of meta-analysis of ketamine gargle prevention in postoperative 24 h sore throat. The square shown for each study (first author and year of publication) is the OR for individual trials, and the corresponding horizontal line is the 95% confidence interval (CI). The diamond is the pooled OR with the 95% CI.

A total of 10 studies reported on the incidence of sore throat 24 h after surgery [1–3, 11, 12, 18–22]. The meta-analysis demonstrated that the ketamine gargle was associated with a significantly reduced occurrence of 24 h sore throat compared to the placebo groups (OR: 0.36; 95% CI: 0.25–0.51; *p* < 0.00001) (Figure 2). There was no significant heterogeneity observed between the studies (*I*^2^ = 0%; *p*=0.44). Following sensitivity analysis, our results remained statistically significant (OR: 0.36; 95% CI: 0.25–0.51) (Supporting Figure 1 in Supporting 3). These findings indicate the robustness of our results.

#### 3.2.2. Secondary Outcome: Subgroup Analysis of Different Time Points Within 24 h

**Figure 3:**
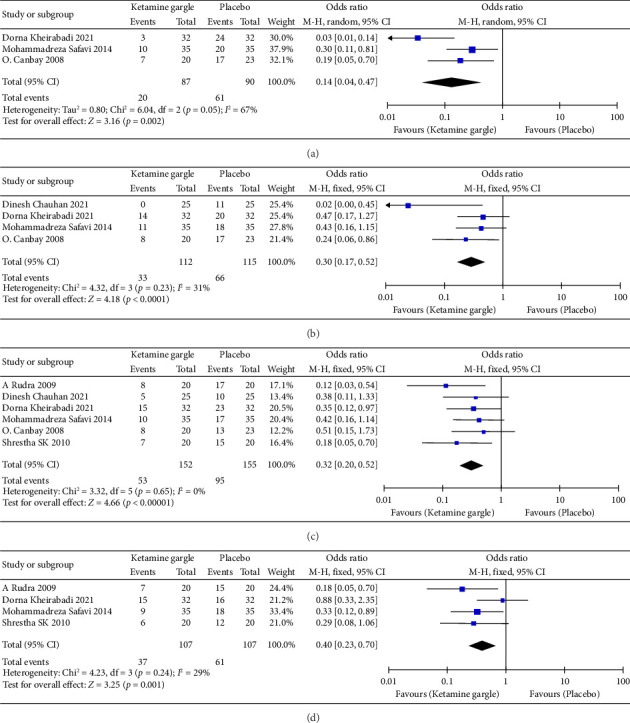
Forest plot of meta-analysis of ketamine gargle prevention in postoperative 0, 2, 4, 8 h sore throat. The square shown for each study (first author and year of publication) is the OR for individual trials, and the corresponding horizontal line is the 95% confidence interval (CI). The diamond is the pooled OR with the 95% CI.

Three studies reported on the occurrence of sore throat immediately after surgery (0 h) [11, 18, 21]. The forest plot demonstrated that the ketamine gargle was effective in reducing POST at 0 h compared to the placebo groups (OR: 0.14; 95% CI: 0.04–0.47; *p*=0.002) (Figure 3(a)). However, there was heterogeneity observed among the studies and potential bias in the results, so cautious interpretation was necessary (*I*^2^ = 67%; *p*=0.05). Four studies included the outcome of sore throat at 2 h postoperatively [3, 11, 18, 21]. The meta-analysis indicated that ketamine gargling significantly reduced the incidence of sore throat at 2 h after surgery (OR: 0.30; 95% CI: 0.17–0.52; *p* < 0.0001) (Figure 3(b)). Furthermore, there was no substantial heterogeneity among the studies, suggesting the reliability of our results (*I*^2^ = 31%; *p*=0.23). Six studies reported on sore throat at 4 h postoperatively [3, 11, 18, 20–22], and the meta-analysis demonstrated that the ketamine gargle was associated with a decreased risk of sore throat at this time point compared to the placebo groups (OR: 0.32; 95% CI: 0.20–0.52; *p* < 0.00001) (Figure 3(c)). No statistically significant heterogeneity was observed among the studies (*I*^2^ = 0%; *p*=0.65), indicating the reliability of our results. Four studies reported on sore throat at 8 h postoperatively [18, 20–22]. Our analysis revealed that ketamine gargle was also associated with a lower risk of sore throat at 8 h after surgery (OR: 0.40; 95% CI: 0.23–0.70; *p*=0.001) (Figure 3(d)). There was no statistically significant heterogeneity observed among the studies (*I*^2^ = 29%; *p*=0.24).

#### 3.2.3. Secondary Outcome: Anesthesia Time

Four studies reported on anesthesia time [2, 18, 20, 21]. Our meta-analysis revealed that ketamine gargle did not result in a significant reduction in anesthesia time (min) (MD: −1.16; 95% CI: −6.44–4.11; *p*=0.67) (Supporting Figure 2 in Supporting 3). Furthermore, there was no notable heterogeneity observed among the studies (*I*^2^ = 0%; *p*=0.89), indicating the reliability of the results.

### 3.3. Risks of Bias and Publication Bias

**Figure 4:**
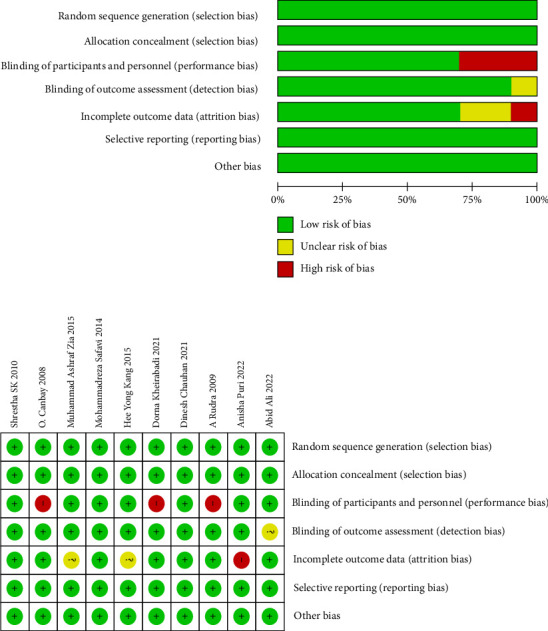
Risk of bias: review of authors' judgments about each risk of bias item for each included study. Green circle: a low risk of bias, yellow circle: an unclear risk of bias, and red circle: a high risk of bias.

Overall, the differences in risk of bias among the studies were minimal. Three studies were classified as having a high risk of bias due to inadequate blinding, while one study had a high risk of bias due to incomplete data [1, 11, 18, 20]. There was a potential risk of bias in three randomized control trials. On the other hand, three randomized control studies were considered to have a low risk of bias and demonstrated good overall quality (Figure 4). The grade ratings for all outcomes are shown in Table 1.

**Figure 5:**
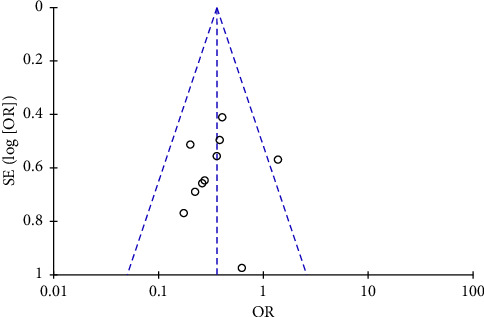
Funnel plot of the studies included in ketamine gargle meta-analysis.

The funnel plot of the included studies in this meta-analysis demonstrated overall symmetry, indicating no evidence of publication bias (Figure 5). Meta-regression based on sample size showed no significant publication bias in this study (*p*=0.86) (Supporting Figure 3 in Supporting 3).

TSA showed that this meta-analysis reached both the traditional and TSA boundaries and was able to definitely obtain significant results (Supporting Figure 4 in Supporting 3).

## 4. Discussion

Our study demonstrated that a prophylactic ketamine gargle is effective in reducing the incidence of POST in surgical patients who require general anesthesia with tracheal intubation, when compared to placebo. This effect may be attributed to ketamine's ability to act as a blocker for various pain-related receptors. Ketamine can block NMDA receptors, as well as 2-amino-3-hydroxy-5-methyl-4-isoxazolepropionic acid and kainic acid receptors in peripheral nerve synapses and the spinal cord [20, 23, 24]. The administration of NMDA receptor antagonists peripherally has been associated with the initiation of the anti-inflammatory cascade and antinociception [25, 26]. The 2-amino-3-hydroxy-5-methyl-4-isoxazolepropionic acid and kainic acid receptors mediate fast excitatory synaptic transmission in the central nervous system [4].

A network meta-analysis conducted by Narinder P. Singh et al. revealed that the topical application of magnesium, followed by liquorice and corticosteroids, was the most effective in inhibiting postoperative throat bleeding 24 h after ETI, while ketamine did not show the same effectiveness [27]. This finding contradicts our own research results. One possible explanation is that most of the ketamine studies included in this network meta-analysis were indirectly compared with other drugs, rather than being directly compared with a placebo. In addition, the network meta-analysis discussed the effect of ketamine on postoperative cough and hoarseness, but the results showed that ketamine did not reduce the incidence of these symptoms 24 h after surgery [27]. This was also a limitation in our study, as only one or two of the ten RCTs included outcome measures such as postoperative cough or hoarseness. Furthermore, a systematic review conducted by Jillian Mayhood et al. demonstrated that ketamine gargling can reduce the incidence of sore throat at 0, 2, 4, 8, and 24 h following airway instrumentation [28]. However, this systematic review only included five RCTs. Despite the consistency between the results of the systematic review and our research, our study is more reliable. Our meta-analysis had a larger sample size, including 10 RCTs with a total of 593 adult participants. In addition, our study encompassed a variety of surgical types, all of which involved ETI, whereas the systematic review by Jillian Mayhood et al. was limited to patients undergoing airway instrumentation.

Ketamine has a short half-life in humans, usually 2–4 min, of which 80% is converted to norketamine by n-demethylation. The half-life of desloratadine is up to 2–4 h [29, 30]. In animal studies, norketamine exerted about one-third of the antiharm perception properties of ketamine [31]. Norketamine may reduce POST within 4 h by reducing the patient's perception of harm. Alternatively, POST may be the result of local trauma leading to sterile mucosal inflammation [11]. Kempe et al. showed oral dryness and inflammation due to mouth breathing in patients undergoing septal surgery [32]. We hypothesized that the presence of sore throat at 24 h postoperatively reflected the slow development of local inflammation. Zhu et al. showed that nebulized ketamine attenuated many of the core components of inflammatory change [26]. Reducing this inflammation by ketamine gargling may be the reason for the reduction of postoperative 24 h sore throat.

Several limitations still exist in this meta-analysis. First, high-risk studies included have the potential to cause bias in the results. Second, the RCTs included in this meta-analysis mainly focused on pelvic or abdominal surgery or septoplasty [23]. Third, only two of the studies we included used low-dose fentanyl for analgesia after surgery [1, 2].

## 5. Conclusion

Our meta-analysis demonstrated the efficacy of a prophylactic ketamine gargle in reducing the incidence of POST across all studied time intervals in patients who required tracheal intubation during general anesthesia, when compared to a placebo. In future research, it is necessary to explore the effect of ketamine gargle on POST in patients who require double-lumen ETI. In addition, further studies should investigate the molecular mechanism through which ketamine reduces POST.

## Figures and Tables

**Table 1:** Quality of evidence rating for all outcomes.

Certainty assessment							No of patients		Effect		Certainty	Importance
No. of studies	Study design	Risk of bias	Inconsistency	Indirectness	Imprecision	Other considerations	Ketamine gargle	Placebo	Relative (95% CI)	Absolute (95% CI)		
*Postoperative 24 h sore throat*												
10	Randomized trials	Not serious	Not serious	Not serious	Not serious	None	89/295 (30.2%)	157/298 (52.7%)	**OR 0.36** (0.25 to 0.51)	**241 fewer per 1000** (from 309 fewer to 165 fewer)	⊕⨁⨁⨁ high	Critical

*Postoperative 8 h sore throat*												
4	Randomized trials	Serious^a^	Not serious	Not serious	Not serious	None	37/107 (34.6%)	61/107 (57.0%)	**OR 0.40** (0.23 to 0.70)	**224 fewer per 1000** (from 336 fewer to 89 fewer)	⨁⨁⨁◯ Moderate^a^	Important

*Postoperative 4 h sore throat*												
6	Randomized trials	Serious^a^	Not serious	Not serious	Not serious	None	53/152 (34.9%)	95/155 (61.3%)	**OR 0.32** (0.20 to 0.52)	**277 fewer per 1000** (from 372 fewer to 161 fewer)	⨁⨁⨁◯ Moderate^a^	Critical

*Postoperative 2 h sore throat*												
4	Randomized trials	Serious^a^	Not serious	Not serious	Not serious	None	33/112 (29.5%)	66/115 (57.4%)	**OR 0.30** (0.17 to 0.52)	**286 fewer per 1000** (from 388 fewer to 162 fewer)	⨁⨁⨁◯ Moderate^a^	Important

*Postoperative 0 h sore throat*												
3	Randomized trials	Serious^a^	Serious^b^	Not serious	Not serious	None	20/87 (23.0%)	61/90 (67.8%)	**OR 0.14** (0.04 to 0.47)	**450 fewer per 1000** (from 600 fewer to 181 fewer)	⨁⨁◯◯ Low^a,b^	Not important

*Anesthesia time*												
4	Randomized trials	Serious^a^	Not serious	Not serious	Not serious	None	107	107	—	**MD 1.16 lower** (6.44 lower to 4.11 higher)	⨁⨁⨁◯ Moderate^a^	Important

*Note:* Author(s): Saihao Fu. Question: Ketamine gargle compared to placebo for postoperative sore throat after tracheal intubation in adults. Setting. Bibliography: No. Effect sizes for outcomes and visualization of the effect of ketamine gargles on outcomes.

Abbreviations: CI, confidence interval; MD, mean difference; OR, odds ratio.

^a^Problems with blinding.

^b^Small sample size.

## Data Availability

All data generated or analyzed during this study are included in this published article and its Supporting information files.

## Supporting Information

Supporting Information 1 Supporting 1: PRISMA 2020 Checklist.

Supporting Information 2 Supporting 2: Search strategy.

Supporting Information 3 Supporting 3: Supporting figures and tables (including Supporting Table 1 and Supporting Figures 1, 2, 3, 4).
