# Supplementary material for: Preoperative Ketamine Gargle for Prevention of Postoperative Sore Throat After Tracheal Intubation in Adults: A Meta-Analysis
Source: Pain Res Manag. 2025 Jan 29;2025:7622696. doi: 10.1155/prm/7622696 (PMC11824847; doi:10.1155/prm/7622696)
Supplement: Supporting Information 2 — Supporting 2: Search strategy. [file 7622696.f2.docx]

**Search strategy**

PubMed: #1"ketamine gargle" #2"postoperative sore throat " OR “sore throat” #3 "Intubation, Intratracheal"[Mesh] OR “Intratracheal Intubation” OR “Intratracheal Intubations” OR “Endotracheal Intubation” OR “Endotracheal Intubations” #4 #1 AND #2 AND #3

Cochrane Library: #1MeSH descriptor: [ketamine gargle] explode all trees:ti,ab,kw #2 MeSH descriptor: [postoperative sore throat] explode all trees OR (sore throat):ti,ab,kw #3MeSH descriptor: [Intubation, Intratracheal] explode all trees OR ((Intratracheal Intubation) OR (Intratracheal Intubations) OR (Endotracheal Intubation) OR (Endotracheal Intubations)):ti,ab,kw #4 #1 AND #2 AND #3

Clinical Trial.gov: “ketamine gargle” and “postoperative sore throat” and “tracheal intubation”

Web of Science:((TS=(ketamine gargle)) AND TS=((postoperative sore throat) OR (sore throat)) AND TS=((Intubation, Intratracheal) OR (Intratracheal Intubation) OR (Intratracheal Intubations) OR (Endotracheal Intubation) OR (Endotracheal Intubations)))

Science Direct: ((ketamine gargle) and ((postoperative sore throat) OR (sore throat)) AND ((Intubation, Intratracheal) OR (Intratracheal Intubation) OR (Intratracheal Intubations) OR (Endotracheal Intubation) OR (Endotracheal Intubations)))

Scopus: (ketamine gargle) and ((postoperative sore throat) OR (sore throat)) AND ((Intubation, Intratracheal) OR (Intratracheal Intubation) OR (Intratracheal Intubations) OR (Endotracheal Intubation) OR (Endotracheal Intubations)) AND (general anesthesia)
