# Supplementary material for: Preoperative Ketamine Gargle for Prevention of Postoperative Sore Throat After Tracheal Intubation in Adults: A Meta-Analysis
Source: Pain Res Manag. 2025 Jan 29;2025:7622696. doi: 10.1155/prm/7622696 (PMC11824847; doi:10.1155/prm/7622696)
Supplement: Supporting Information 3 — Supporting 3: Supporting figures and tables (including Supporting Table 1 and Supporting Figures 1, 2, 3, 4). [file 7622696.f3.docx]

Supplemental Table 1: Baseline characteristics

| Name year | Country | K-n | P-n | K-age (yrs.) | P-age (yrs.) | K-male | P-male | ASA | Doses of ketamine used | Methods | Type of surgery | Size of tracheal tube |
| --- | --- | --- | --- | --- | --- | --- | --- | --- | --- | --- | --- | --- |
| ABID ALI 2022 | pakistan | 43 | 43 | 38.58±7.57 | 38.21±7.33 | 30 | 29 | I-II | Ketamine 50mg in 29 mL drinking water Gargling for 40 seconds | Four-point scale | pelvic/abdominal elective surgery | Female: 7.5mm, Male: 8mm |
| O. Canbay 2008 | Turkey | 20 | 23 | 26.46±5.08 | 23.71±3.11 |  |  | I-II | Ketamine 40mg in 30 mL drinking water Gargling for 30 seconds | Four-point scale | septorhinoplasty | Female: 7-8mm, Male: 8-9mm |
| DINESH CHAUHAN 2021 | India | 25 | 25 | 32.76±8.24 | 32.72±9.43 | 14 | 8 | I-II | Ketamine 50mg in 29 mL normal saline Gargling for 30 seconds | Four-point scale | Not specific | Female: 7-7.5mm, Male: 8-8.5mm |
| Hee Yong Kang 2015 | Korea | 20 | 20 | 42.3 ± 11.9 | 42.5 ± 11.7 | 9 | 9 | I-II | Ketamine 50mg in 29 mL normal saline Gargling for 30 seconds | Four-point scale | laparoscopic cholecystectomy | Female: 7mm, Male: 7.5mm |
| Dorna Kheirabadi 2021 | Iran | 32 | 32 | 35.12±12.26 | 32.84±13.50 | 24 | 16 | I-II | Ketamine 50mg in 29 mL normal saline Gargling for 30 seconds | Four-point scale | septorhinoplasty | Female: 7mm, Male: 8mm |
| Anisha Puri 2022 | India | 30 | 30 |  |  |  |  | I-II | Ketamine 40mg in 30 mL normal saline Gargling | Four-point scale | ear surgeries | Female: 7mm, Male: 8mm |
| A Rudra 2009 | India | 20 | 20 | 37.5 ±12.5 | 36.7 ± 12.3 | 11 | 8 | I-II | Ketamine 50mg in 29 mL normal saline Gargling for 30 seconds | Four-point scale | abdominal and pelvic surgery | Female: 7.5mm, Male: 8.5mm |
| Mohammadreza Safavi 2014 | Iran | 35 | 35 | 31.1±13.6 | 34.5±13.4 | 26 | 32 | I-II | Ketamine 40mg in 30 mL normal saline Gargling for 30 seconds | Four-point scale | Not specific | Female: 7mm, Male: 8mm |
| Shrestha SK 2010 | Nepal | 20 | 20 | 36.8 ± 12.3 | 32.9± 7.7 | 5 | 6 | I-II | Ketamine 50mg in 30 mL normal saline Gargling for 30 seconds | Four-point scale | abdominal and orthopedics surgery |  |
| MUHAMMAD ASHRAF ZIA 2015 | pakistan | 50 | 50 | 31.54±8.15 | 29.74±8.40 | 33 | 35 | I-II | Ketamine 50mg in 29 mL normal saline Gargling for 30 seconds | Four-point scale | septorhinoplasty | Female: 7mm, Male: 7.5mm |

Abbreviations: K-n: number of people in the ketamine gargle group; P-n: number of people in the placebo group; K-age: Age of patients in the ketamine gargle group; P-age: Age of patients in the placebo group; K-male: Number of male patients in the ketamine gargle group; P-male: Number of male patients in the placebo group; ASA: American society of Anesthesiologists (ASA) physical status classification system.

Supplemental Fig.1: Sensitivity analysis of ketamine gargle prevention in postoperative 24h sore throat.


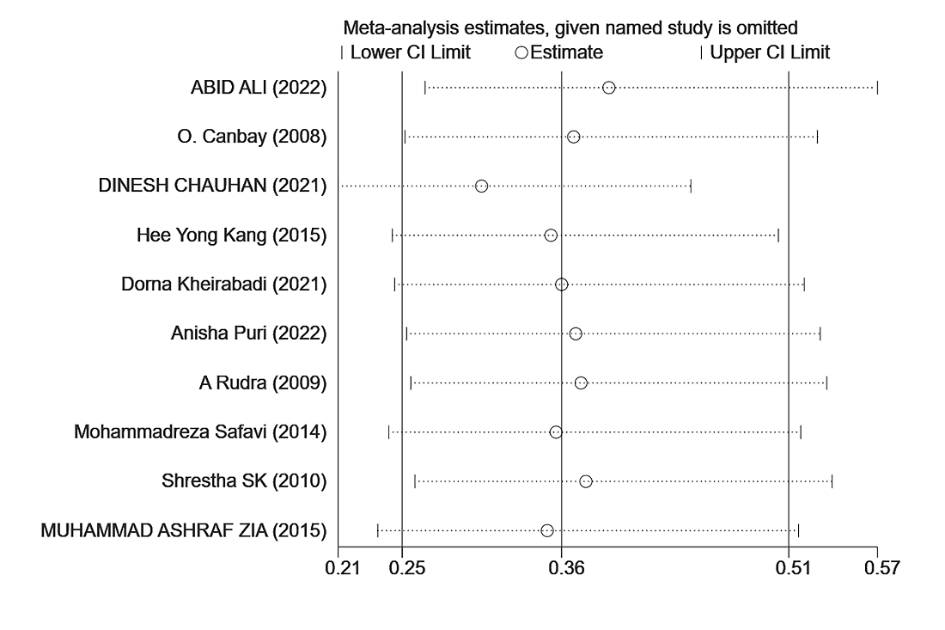


Supplemental Fig.2: Forest plot of meta-analysis of ketamine gargle reduction in anesthesia time. The square shown for each study (first author and year of publication) is the OR for individual trials, and the corresponding horizontal line is the 95% confidence interval (CI). The diamond is the pooled OR with the 95% CI.





Supplemental Fig.3: Meta-regression based on sample size


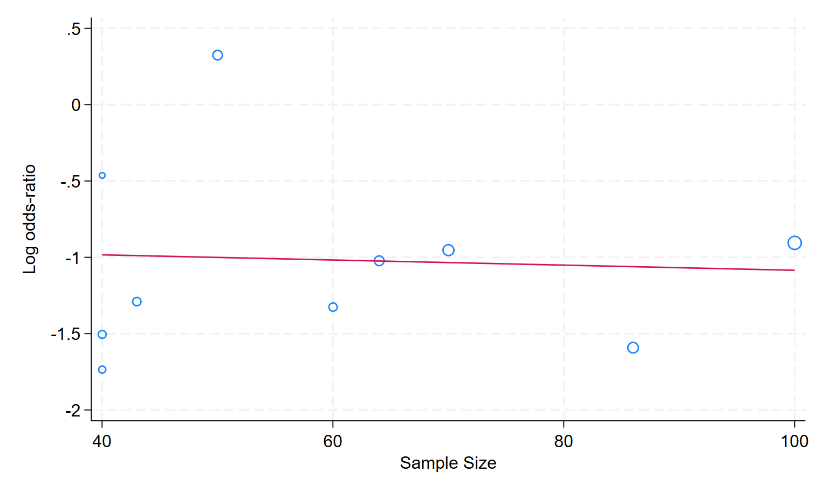


Abbreviations: *P* = 0.86, indicating that sample size is not a source of heterogeneity.

Supplemental Fig.4: Trial sequential analysis for this meta-analysis


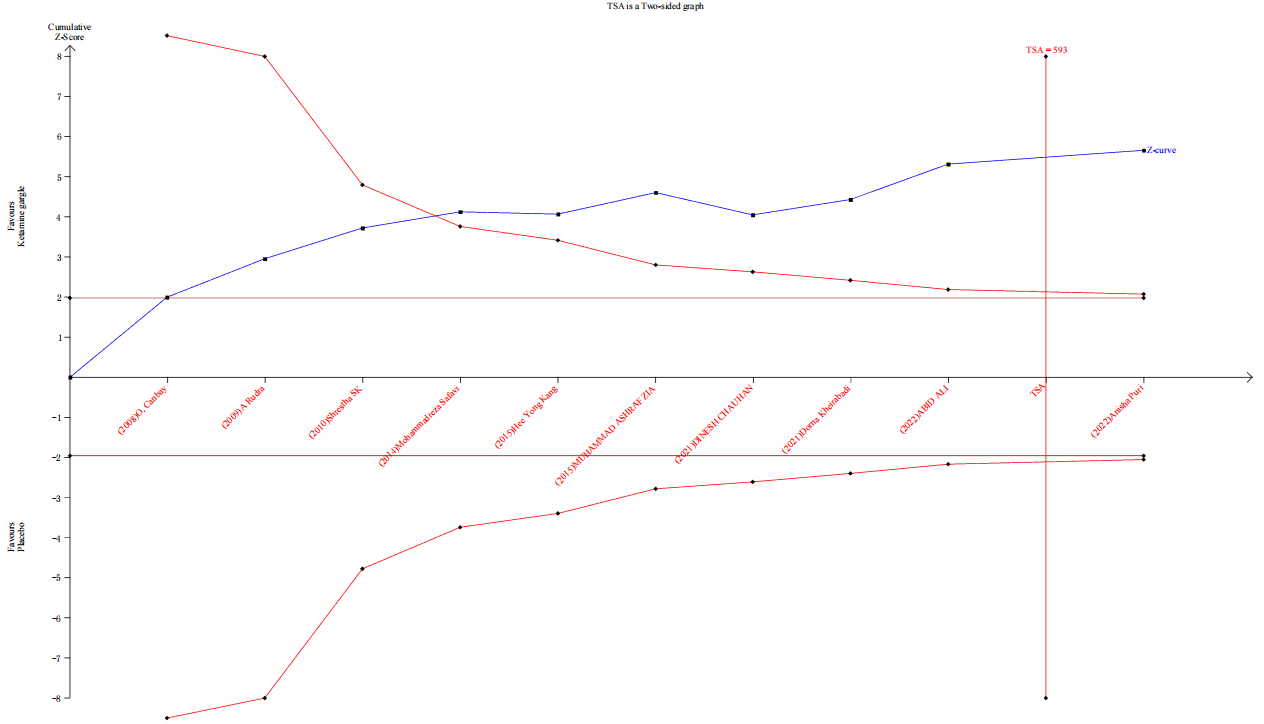


Abbreviations: The red dash represents the TSA boundary value; The red horizontal line represents the traditional boundary value (Z = 1.96); The blue dash represents the results of this meta-analysis; Vertical lines represent RIS; When the curve crosses both the traditional and TSA boundaries, it indicates that a positive result can be obtained that does not require the inclusion of further studies to be analyzed.
